# Carbon Nanoparticles Functionalized with Carboxylic Acid Improved the Germination and Seedling Vigor in Upland Boreal Forest Species

**DOI:** 10.3390/nano10010176

**Published:** 2020-01-20

**Authors:** Md. Hossen Ali, Jean-Marie Sobze, Thu Huong Pham, Muhammad Nadeem, Chen Liu, Lakshman Galagedara, Mumtaz Cheema, Raymond Thomas

**Affiliations:** 1 School of Science and the Environment/Boreal Ecosystem Research Facility, Grenfell Campus, Memorial University of Newfoundland, Corner Brook, NL A2H 5G5, Canada; mhali@grenfell.mun.ca (M.H.A.); tpham@grenfell.mun.ca (T.H.P.); cliu@grenfell.mun.ca (C.L.); lgalagedara@grenfell.mun.ca (L.G.); mcheema@grenfell.mun.ca (M.C.); 2 Northern Alberta Institute of Technology, 8102-99 Avenue, Peace River, AL T8S 1R2, Canada; jeanmars@nait.ca

**Keywords:** carbon nanoparticles (CNPs), germination rate, membrane lipid, seed dormancy, seedling vigor

## Abstract

Nanopriming has been shown to significantly improve seed germination and seedling vigor in several agricultural crops. However, this approach has not been applied to native upland boreal forest species with complex seed dormancy to improve propagation. Herein, we demonstrate the effectiveness of carbon nanoparticles functionalized with carboxylic acids in resolving seed dormancy and improved the propagation of two native upland boreal forest species. Seed priming with carbon nanoparticles functionalized with carboxylic acids followed by stratification were observed to be the most effective in improving germination to 90% in green alder (*Alnus viridis* L.) compared to 60% in the control. Conversely, a combination of carbon nanoparticles (CNPs), especially multiwall carbon nanoparticles functionalized with carboxylic acid (MWCNT–COOH), cold stratification, mechanical scarification and hormonal priming (gibberellic acid) was effective for buffaloberry seeds (*Shepherdia canadensis* L.). Both concentrations (20 µg and 40 µg) of MWCNT–COOH had a higher percent germination (90%) compared to all other treatments. Furthermore, we observed the improvement in germination, seedling vigor and resolution of both embryo and seed coat dormancy in upland boreal forest species appears to be associated with the remodeling of C18:3 enriched fatty acids in the following seed membrane lipid molecular species: PC18:1/18:3, PG16:1/18:3, PE18:3/18:2, and digalactosyldiacylglycerol (DGDG18:3/18:3). These findings suggest that nanopriming may be a useful approach to resolve seed dormancy issues and improve the seed germination in non-resource upland boreal forest species ideally suited for forest reclamation following resource mining.

## 1. Introduction

Several factors play key role in determining seed germination, early seedling establishment and adaptation to diverse set of environmental conditions [1,2,3]. Seed dormancy is a key player in successful seedling establishment and is defined as a state whereby seeds are unable to germinate in conditions suitable for germination [4]. Seeds have three main types of dormancy: (a) Hard seed coats where seeds are impermeable to water and gases (physical dormancy). As such, water uptake and oxygen exchange are restricted and such dormancy can only be alleviated by creating fissures in the seed coat via mechanical or chemical methods. (b) The second is embryo or internal dormancy where embryo cells stop growing and it is caused by endogenous hormones such as cytokinin, indole-3-acetic acid and abscisic acid. (c) The 3rd main seed dormancy is caused by germination inhibitors such as cis-ABA, β-d-glucopyranosyl ester, benzoic acid, salicylic acid, chlorogenic acid, coumarin, etc., which inhibit seed germination and plant growth [5]. Many of the native non-resource upland boreal forest species have seed dormancy issues that limit their propagation. In addition, there is limited knowledge surrounding what kind of dormancies are present in these native species, and what suitable approach or seed treatments may be applicable to break these dormancies.

There is high interest in the propagation of non-resource species for upland boreal forest restoration, revegetation or reclamation arising from anthropogenic disturbances. For example, oil and gas mining resulted in forest fragmentation due to the construction of cut lines, roads, electrical lines, drill sites, etc. [6]. Natural resource extraction companies, for instance oil and gas companies, are mandated by law to return the ecologically important disturbed sites back to a functional upland boreal forest ecosystem, using native plant species [6,7,8]. Buffaloberry (*Shepherdia canadensis* L.) is a small shrub of the genus *Shepherdia* that produce edible berries. The plant is described as a deciduous shrub, growing to a maximum of 1–4 m, with 4–8 mm long seeds, and have both seed coat and embryo dormancy [9,10]. Buffaloberry is native to the boreal ecosystem in Northern America, including the Prairies in Canada, as well as eastern province (Newfoundland and Labrador). They are also important indicator species in upland boreal forest ecosytems. Therefore, buffaloberry was selected for current study as an important indicator species that could be used to assess performance during reclamation of anthropogenically disturbed sites in boreal upland ecosystem. Another upland species of importance in the boreal forest landscape is green alder (*Alnus viridis* L.), due to its nitrogen-fixing abilities in boreal forest soils [11]. These are large (3–12 m tall) shrubs of the genus *Alnus*. The seeds of green alder are small, 1–2 mm long, light brown, with a narrow encircling wing, and has embryo dormancy [12]. 

Different priming techniques have been applied in agriculture crops to alleviate seed dormancy, improve germination rates, enhance seedling vigor and seedling capacity to grow and establish across a range of sites with varying environmental conditions [13,14,15,16,17,18,19]. Such priming techniques include hydrating seeds in salt solutions (halopriming), solutions of beneficial microbes (biopriming), osmotic solution (osmopriming), plant hormones solutions (hormonal priming), presence of a magnetic field (magneto-priming), solutions mixed with a solid carrier (matriconditioning), solutions of nanoparticles (nanopriming) or hydrating seeds in water (hydropriming) [15,20,21]. The realization of the potential use of nanotechnology in many sectors, including agriculture, has increased recently [18,22,23]. The use of nanoparticles in enhancing seed germination, plant growth, and to modulate how plants interact with their environment at both, the cellular or molecular level has been demonstrated in several recent studies [18,23,24,25,26]. For instance, CNPs applications improve the germination rates in various plant species [17,22,24,27,28,29,30]. The ability of CNPs to traverse both the cell wall and seed coat have been proposed as a possible mechanism through which the increase germination rates have been achieved [19,24,31,32,33]. Hopbush (*Dodonaea viscosa* L.) seeds primed with multi-walled carbon nanotubes (MWCNTs) resulted in dramatic improvements in the seed germination rates and seedling vigor [34]. Consistent with the observations of enhancing germination percentages and seedling vigor reported in agriculture species primed with different nanoparticles [18,23,24,28,35,36], the possibility exists that this approach could be suitable to resolve the low germinations observed for many non-resource boreal upland forest species such as green alder and buffaloberry.

Cold stratification, incubation with gibberellic acid (hormone priming) and seed coat scarification has been shown to be successful in enhancing the germination rate of rosemary seeds having hard seed coat, embryo, and physiological seed dormancy [12,37,38,39,40]. This could be a useful approach to apply to seeds such as buffaloberry, which also has multiple seed dormancies. There are many studies in the scientific literature demonstrating the beneficial effects of nanotechnology in agricultural species to address issues with seed dormancy and low germination [18,19,21,24,28,30,41,42]. However, to the best of our knowledge, very few studies have been done to assess the effects of nanopriming [17,21,43], particularly in combination with stratification and hormone priming in resolving seed dormancy in native non-resource upland boreal forest species, such as buffaloberry and green alder.

During germination, a seed goes through various physiological (water uptake, respiration, etc.) and biological (lipids, proteins, etc.) alterations [44,45,46,47,48]. Changes in seed membrane lipid metabolism have been reported to be highly correlated with improved seed germination [49]. For example, during *Medicago sativa* germination, it has been reported that membrane lipids such as phospholipids, galactolipids, and sulfolipids increased several folds. In fact, phosphatidylcholine (PC) was observed to increase several folds compared to other phospholipids, particularly linoleic acid enriched PC molecular species [50]. Changes in seed fatty acids have also been reported to be directly associated with resolving embryo dormancy in *Amaranthus albus* L. [51]. Thus, we hypothesize that seed priming with CNPs can be used to overcome seed dormancy issues in upland boreal forest species by modulating the membrane lipid metabolism. Herein we seek to investigate whether nanopriming with CNPs could be useful for breaking seed dormancy, improve germination and seed vigor in two native upland boreal forest species.

## 2. Material and Methods

The experiment was undertaken in the Boreal Ecosystems Research Facility at Grenfell Campus, Memorial University of Newfoundland, Corner Brook, Canada. Seeds of two upland boreal forest species; buffaloberry (*Shepherdia canadensis* L.) and green alder (*Alnus viridis* L.) were obtained from the Northern Alberta Institute of Technology (NAIT) and stored at −20 °C after receiving, until the start of experiments. The experimental design consisted of five treatments: (i) multiwall carbon nanoparticles; MWCNT, (ii) MWCNT functionalized with carboxylic acid (MWCNT–COOH) (iii) graphene, (iv) up-conversion nano-phosphorous (Up-con. NP), and (v) deionized water (DI) as the control.

For each treatment, a total of 25 buffaloberry and green alder seeds were primed overnight in glass vials containing either 20 µg/mL or 40 µg/mL CNPs dissolved in DI water following sonication to get the nanoparticles in solution. Three vials, each containing 25 seeds for each treatment, were prepared for a total of three replicates per treatment. After priming, seeds were sown in condensation free Petri plates (P5481, Sigma-Aldrich, Oakville, ON, Canada) lined with moist germination grade Whatman filter paper (CA28297-216, VWR, Mississauga, ON, Canada). This batch was incubated at room temperature on germination racks illuminated with LED lights for 3 weeks, and germination recorded daily. DI water (1 mL) was sprayed to keep filter papers moist, as required throughout the germination evaluation period for all treatments. The second batch of buffaloberry and green alder seeds (with the same priming procedure as the first batch) were sown in condensation free Petri plates and incubated in a fridge at 2–4 °C for the cold stratification treatment. After 15 days of stratification, all Petri plates containing green alder and buffaloberry seeds were incubated at room temperature on germination racks under continuous LED lighting (24 h) during germination evaluations. Both seeds were tested in two treatments, as outlined below:

CNPs: Imbibition for 24 h with select CNP and then incubated at room temperature.

CNPs + Stratification: Imbibition for 24 h with select CNPs and then incubate all Petri plates in fridge (2–4 °C) for 15 days followed by incubation at room temperature until all seeds germinated or were decayed.

In the case of buffaloberry, two additional experimental treatments were conducted to evaluate the difference between mechanical and chemical scarifications, along with plant hormone treatments on germination. During mechanical scarification, CNPs were added along with gibberellic acid @ 1000 mg/L. Mechanical scarification was carried out using sandpaper (Mastercraft) bought from a local hardware store by gently rubbing the surface of the seeds between the sheets of sandpaper to scratch the seed surface. Following scarification, seeds were primed with and without nanoparticles and hormone. In the chemical scarification, CNPs, gibberellic acid (1000 mg/L), and 80% sulfuric acid were added. Chemical scarification was made by immersing 25 seeds of buffaloberry from each replication in a beaker with concentrated sulphuric acid for 5 min, stirring with a glass rod. After draining the acid, the seeds were soaked in water for 1 h followed by 1 min washing in running tap water, then surface dried with blotting paper [52,53]. After chemical scarification, the seeds were primed overnight in glass vials with each CNPs (20 µg/mL and 40 µg/mL) and DI water (control). All the Petri plates were incubated at 2–4 °C (cold stratified) for 120 days. A randomized approach was used to carry out the experiments with three replications per treatment (25 seeds each replication). In summary, the two additional treatments for buffaloberry were as follows: 

### 2.1. Mechanical Scarification + Stratification + CNPs + Gibberellic Acid (GA)

First, mechanical scarification was done by sandpaper. Seeds were imbibed for 24 h with selected CNPs (MWCNT–COOH, MWCNT, and Graphene) using two concentrations (20 µg/mL and 40 µg/mL) followed by GA (1000 mg/L). Cold stratification was performed in a refrigerator (2–4 °C) for 120 days, then seeds germinated at room temperature for an additional 120 days.

### 2.2. Chemical Scarification + Stratification + CNPs + GA

Chemical scarification was done with both 80% and 90% sulphuric acid. Seeds were imbibed for 24 h with select CNPs (MWCNT–COOH, MWCNT, and Graphene) followed by GA (1000 mg/L). Cold stratification was performed in a refrigerator (2–4 °C) for 120 days, then seeds germinated at room temperature. 

### 2.3. Tetrazolium Test

The tetrazolium technique was used to test and evaluate the viability of seeds in a seed lot [54] prior to germination. For the tetrazolium test, 20 seeds for each replication were imbibed in DI water with and without each nanoparticle for 6 h at 25 °C [54]. Seeds were then dissected longitudinally through the embryo with a scalpel, and half were placed in tetrazolium solution (2,3,5-Triphenyltetrazolium chloride) for 6 h at a concentration of 0.050% (w/v) as described by Paiva et al., [54]. Samples were then rinsed with DI water to remove excess stain. The stained seeds were then evaluated under low power (0.5×) using a light microscope (Nikon SMZ1500, Valley Microscopes, Melville, NY, USA). Viable seed stained bright red, while pink and very dark red stains are indicative of dead tissue. As such, seeds were classified into three categories based on the following staining patterns: completely stained, completely unstained, and partially stained [55].

### 2.4. Electrical Conductivity Test

Electrical conductivity measures the leaked electrolytes from the membrane and gives an estimation of membrane permeability. The electrical conductivity of seeds from each treatment was measured using an EC meter (Field Scout EC Meter, Spectrum Technologies, Inc., Aurora, IL, USA). Briefly, seeds were soaked overnight in CNPs or DI water (control) and readings for each treatment were recorded and reported as milli-Siemens per centimeter (mS/cm). Low EC measurements are indicative of seeds with high vigor [56]. For each concentration of CNPs, there were three replications and 25 seeds per replication.

### 2.5. Evaluation of Normal and Abnormal Seedlings

Seedlings were classified into abnormal (ANS) and normal (NS) depending upon the germination tests using the International Seed Testing Association (ISTA) handbook on seedling evaluation procedures [53]. ANS were determined if the roots were stunted, stubby, missing, broken, or split from the tip, as well as having shoots which were short and thick, split, constricted, twisted, decayed and the leaves deformed, damaged, or missing [53].

### 2.6. Germination Percentages

To measure seed germination percentage and seedling vigor, seeds were observed daily throughout the experimental period. The seed germination rate was recorded in each treatment by counting the number of emerged seeds each day, as well as the total seeds germinated. Three-day old seedlings were then harvested to observe the emerged seedling lipids. Shoot and root length were measured before storing at −80 °C. Germination percentage was calculated as:

(1) $$\mathrm{Germination}\;\left(\%\right)=\;\frac{\mathrm{Number}\;\mathrm{of}\;\mathrm{germinated}\;\mathrm{seeds}}{\mathrm{Number}\;\mathrm{of}\;\mathrm{total}\;\mathrm{seeds}}\times 100$$

### 2.7. Seedling Vigor Index (SVI)

After three days of germination, seedlings were removed from Petri plates, and the seedling length (cm) was measured using a ruler. The seedling vigor index (SVI) was calculated as follows:

(2) $$\mathrm{SVI}=\;\frac{\mathrm{Seedling}\;\mathrm{length}\;\left(\mathrm{cm}\right)}{100}\;\times \%\;\mathrm{germination}$$

### 2.8. Lipid Extraction and Analyses

#### 2.8.1. Chemicals 

High-performance liquid chromatography (HPLC) grade acetonitrile, chloroform, and methanol were purchased from Fisher Scientific (Hampton, NH, USA). DI water was obtained from the PURELAB Purification System (ELGA Labwater, ON, Canada). HPLC grade acetic acid, formic acid, and ammonium acetate were purchased from Sigma-Aldrich (Oakville, ON, Canada). Commercial standards of phospholipids were purchased from Avanti Polar Lipids (Alabaster, AL, USA).

#### 2.8.2. Lipid Extraction

Germinated seedlings from each replicate per treatment were collected and then incubated in hot isopropanol for 15 min, following homogenization with an OMNI tissue homogenizer (Tissue Master 125, OMNI International, Kennesaw, GA, USA). The homogenate was used for further lipid extraction and analysis by adapting and updated version of the Bligh and Dyer method was used for lipid extraction [57]. A total of 10 mg of the sample was transferred to glass centrifuge tubes, and the following solvents were added: 1 mL of methanol containing 0.01% butylated hydroxytoluene (BHT), 1 mL chloroform and 0.8 mL water. The sample with the solutions was homogenized again using an OMNI tissue homogenizer. The sample mixture was centrifuged at 5000 rpm for 15 min. After centrifugation, the organic layer (bottom of the vial) containing the lipids were transferred to pre-weighed 4 mL sample vials with PTFE lined caps (VWR, Mississauga, ON, Canada). The organic layer was dried under a nitrogen stream, and then weighed to determine the amount of lipids recovered [57]. The recovered lipids in each vial were re-suspended in 1 mL chloroform:methanol (1:1 v/v) and stored at −20 °C for further analysis.

### 2.9. Plant Membrane Lipids Analysis Using UHPLC-C30RP-HESI-HRMS/MS

Ultra-high-performance liquid chromatograph (UHPLC), coupled to a C30 reverse phase chromatography column and heated electrospray ionization high-resolution tandem mass spectrometry (UHPLC-C30RP-HESI-HRMS/MS), was used to separate the plant membrane lipid classes and molecular species as described in our previous works [58,59,60]. The lipid analyses were carried out using a Q-Exactive Orbitrap mass spectrometer (Thermo Scientific, Berkeley, MO, USA) linked with an automated Dionex UltiMate 3000 ultra-high-performance liquid chromatograph system controlled by Chromeleon software. An Accucore C30 column (150 × 2 mm^2^ I.D., particle size: 2.6 µm, pore diameter: 150 Å), purchased from ThermoFisher Scientific (Mississauga, ON, Canada), was used for lipid separation. The solvent scheme used to resolve all the complex lipids was as follows: Solvent A contained water (60:40 v/v) with 10 mM ammonium formate and 0.1% formic acid: acetonitrile. Solvent B contained H_2_O with 10 mM ammonium formate and 0.1% formic acid:isopropanol:acetonitrile (90:10:1 v/v/v).

UHPLC-C30RP chromatography separation was conducted at 30 °C (column oven temperature) with a flow frequency of 0.2 mL/min, and 10 µL of the sample extract suspended in methanol (2:1 v/v): chloroform was injected in the machine. The following gradient was used for separating the different lipid classes and molecular species: 30% B solvent for 3 min, increased to 43% over 5 min, then to 50% for 1 min, then to 90% over 9 min, then to 99% over 8 min, and lastly at 99% for 4 min. The column was re-equilibrated to starting conditions (70% solvent A) for 5 min before each new injection. Full scan HESI-MS and MS/MS acquisitions were done on a Q-Exactive Orbitrap mass spectrometer operated in both positive and negative modes, and the machine controlled by X-Calibur software 4.0. The following parameters were used for the Orbitrap mass spectrometer, sheath gas: 40, auxiliary gas: 2, ion spray voltage: 3.2 kV, capillary temperature: 300 °C; S-lens RF: 30 V; mass range: 200–2000 m/z; full scan mode at a resolution of 70,000 m/z; top-20 data-dependent MS/MS at a resolution of 35,000 m/z and collision energy of 35 (arbitrary unit); an injection time of 35 min for C30RP chromatography; isolation window: 1 m/z; automatic gain control target: 1e5 with a dynamic exclusion setting of 5 s. The machine was adjusted to 1 ppm using ESI positive and negative calibration solutions (Thermo Scientific, Berkeley, MO, USA). Tune parameters were optimized using PC 18:1(9Z)/18:1(9Z), Cer d18:1/18:1(9Z), PG 18:1 (9Z)/18:1(9Z), SQDG 18:3(9Z,12Z,15Z)/16:0, MGDG 18:3(9Z,12Z,15Z)/16:3(7Z,10Z,13Z) and DGDG 18:3(9Z,12Z,15Z)/18:3(9Z,12Z,15Z) lipid standards (Avanti Polar Lipids, Alabaster, AL, USA) in positive and negative ion modes.

### 2.10. Data Processing 

All lipidomics data were acquired and processed using X-Calibur 4.0 Thermo Scientific, Berkeley, MO, USA) and LipidSearch version 4.1 (Mitsui Knowledge Industry, Tokyo, Japan) software packages. LipidSearch was used for the identification and quantification of the lipid classes and lipid molecular species. XLSTAT (Addinsoft, Paris, France) was used for statistical analysis. Relationships between seedling vigor, germination, and seed membrane lipidome were assess using redundancy analysis (RDA) and Pearson’s correlation coefficient. Treatments clustering in distinct quadrants of the biplot following RDA were analysed using ANOVA. Where treatment effects were different, means were separated using Fishers LSD at α = 0.05. Figures were created with Sigma plot 13 (Systat Software Inc., San Jose, CA, USA).

## 3. Results

### 3.1. Seed and Seedling Quality Analysis

**Figure 1:**
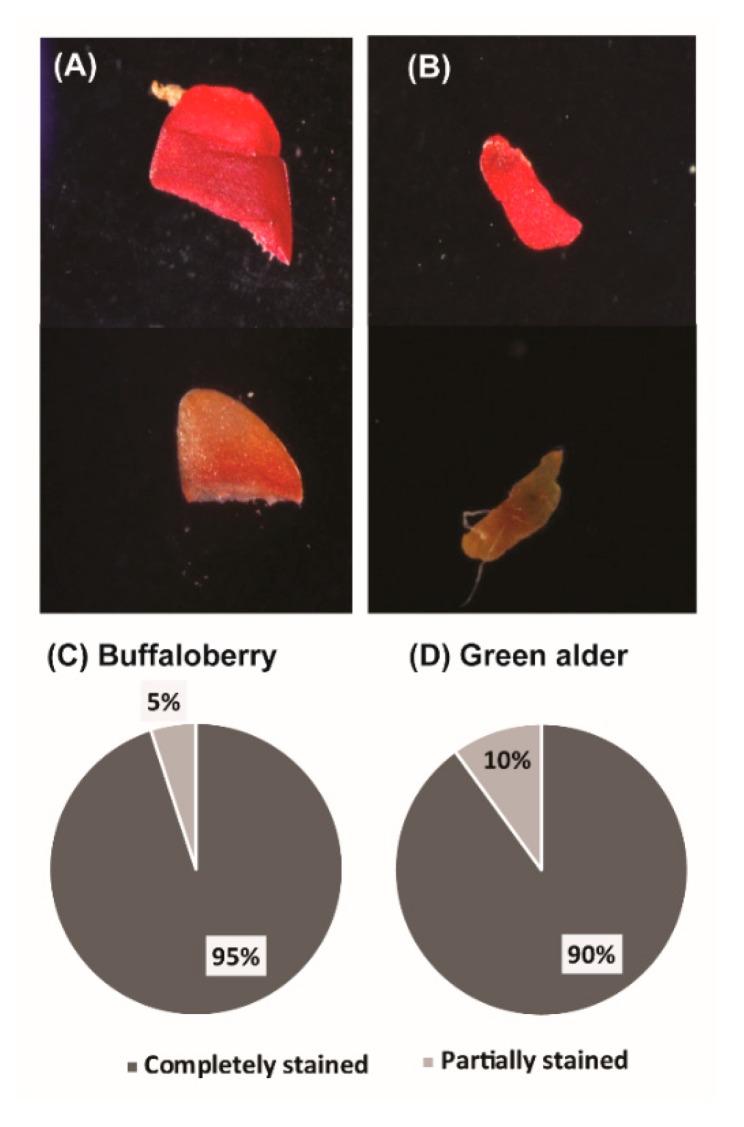
Tetrazolium test results showing viability of seeds following treatments. Stained and unstained seed of buffalo berry (**A**), stained and unstained seed of green alder (**B**) and percentages of completely stained (**C**), and partially stained seeds (**D**).

The tetrazolium test was used to evaluate seed viability. In this test, viable tissues of seeds stained bright red color, and seeds that were dead or not viable stained dark red, pink, or no color at all (Figure 1A,B). For both green alder and buffalo berry species, 90% and 95% of the seeds were observed to be viable seeds, respectively (Figure 1C,D), as they stained bright red in color. In contrast, 10% and 5% of the seeds were partially stained for buffaloberry and green alder respectively (Figure 1C,D).

**Figure 2:**
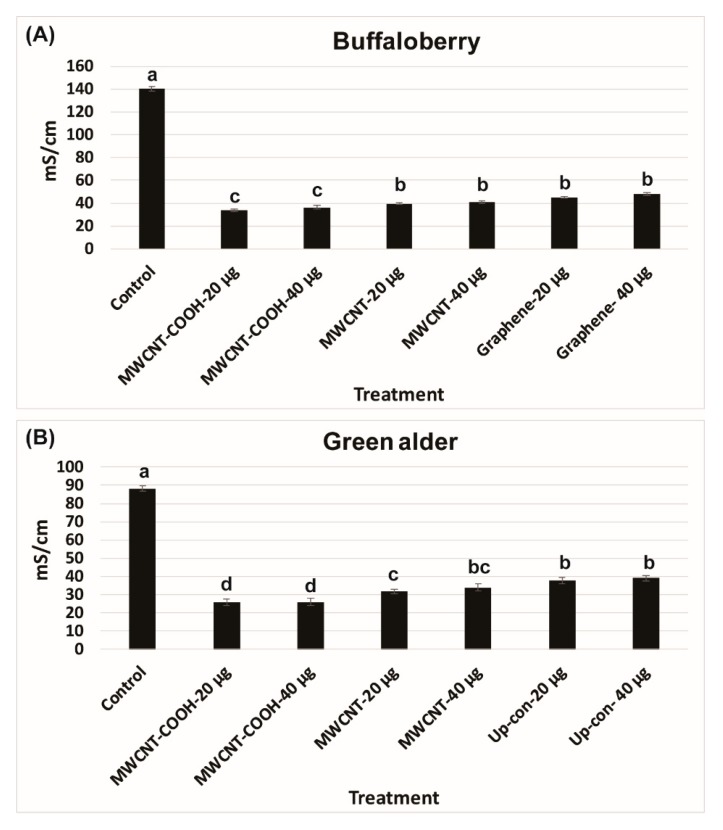
Electrical conductivity (mS/cm) test in buffaloberry (**A**) and green alder (**B**) upland boreal forest species. Values in bar chart represent means ± standard errors. Letters indicate significant difference at α = 0.05. Control = no CNPs added. MWCNT–COOH = multiwall carbon nanotubes functionalized with carboxylic acid, MWCNT = multiwall carbon nanotubes and Up-con = up-conversion nanophosphors.

**Figure 3:**
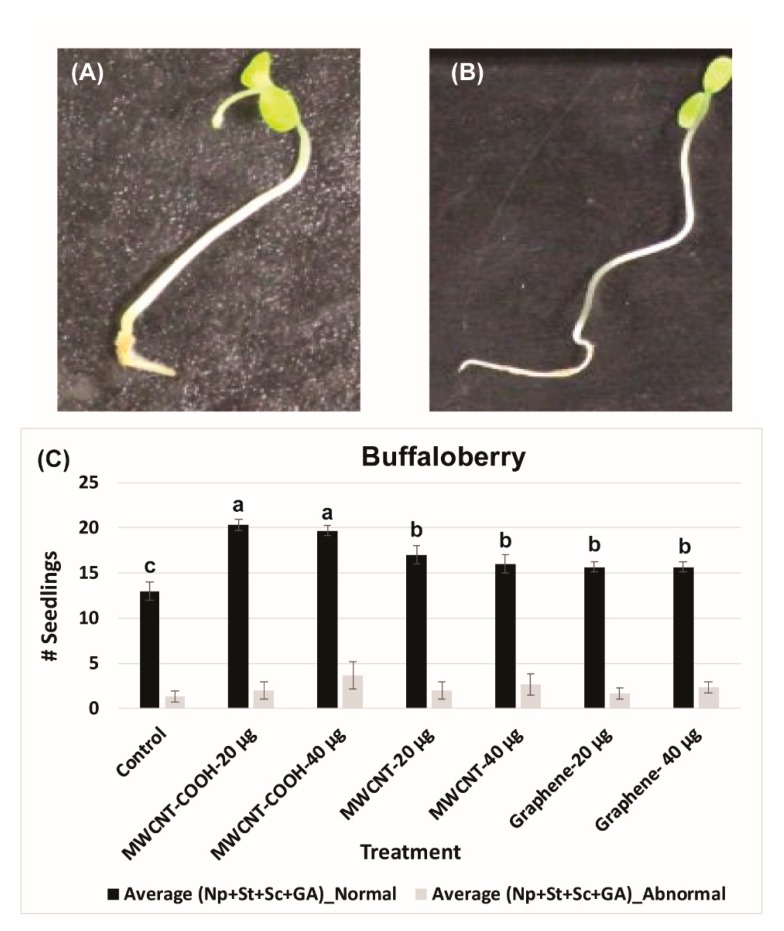
Abnormal (**A**) and normal (**B**) buffaloberry seedlings observed during current study. Effect of different treatments on total number of normal and abnormal seedlings in buffaloberry (**C**). Each bar presents total number of seedlings ± standard errors, whereas bars sharing different letters are significant different from each other at α = 0.05. Control = no CNPs added, MWCNT–COOH = multiwall carbon nanotubes functionalized with carboxylic acid, MWCNT= multiwall carbon nanotubes, UP-con NP = up conversion nanophosphors, N = 100 plants per treatment.

The lower electrical conductivity measurements in the solution following seed priming, the higher is the seed vigor. An EC of 140 mS/cm was recorded for buffaloberry species in the control, which was higher than all the nanoprimed treatments (Figure 2A). In contrast, the EC reading was very low in all the nanoprimed treatments compared to the control. The electrical conductivity measurements in the primed seeds ranged from 35 to 40 mS/cm in all treatments, demonstrating the seeds were viable before seed germination evaluations. Similar trends were also found in the case of green alder species (Figure 2B). In the control, the EC was observed to be 90 mS/cm compared to the CNPs treatments, where it was lower ranging in values, between 25 to 35 mS/cm (Figure 2B). Approximately 85% of the seedlings were normal in green alder primed with MWCNT–COOH (both 20 µg/mL and 40 µg/mL), and this was significantly higher than the control and other nanoprimed treatments (Figure 3).

**Figure 4:**
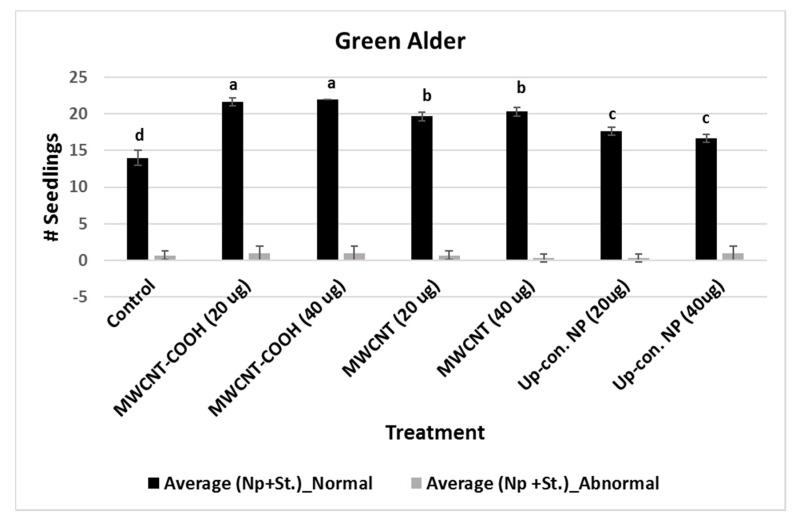
Effects of different priming treatments on number of normal and abnormal seedlings in green alder. Each bar represents the number of seedlings ± standard errors. Different letters on vertical bars indicate significant differences at α = 0.05. Control = no CNPs added, MWCNT–COOH = multiwall carbon nanotubes functionalized with carboxylic acid, MWCNT = multiwall carbon nanotubes, UP-con NP = up conversion nanophosphors, N = 100 plants per treatment.

For MWCNT (20 µg/mL and 40 µg/mL) and up conversion nanophosphorus (20 µg/mL and 40 µg/mL), the NS were 80% and 70%, respectively, while in the control it was 55%. Evaluation of normal and abnormal seedlings was done only in the CNPs with stratification treatment because the germination rate was very low for the CNPs without stratification treatment. The abnormal seedling percentages did not show much difference between the treatments and the control. In the case of buffaloberry, evaluation of normal and abnormal seedlings was done only in seeds primed with nanoparticles, hormone (GA) treated, mechanically scarified, and stratified at 2 to 4 °C. Both concentrations (20 and 40 µg/mL) of MWCNT–COOH primed seeds had higher percent of normal seedlings (80%) compared to the control and other nanoprimed treatments. The normal seedlings were 65%, 60%, and 50% for MWCNT, graphene, and control, respectively (Figure 4). For MWCNT at 20 µg/mL concentration, it was 70% compared to 65% at 40 µg/mL. Seeds nanoprimed with graphene had 60% NS seedlings for both concentrations. In the control, the percentages of normal and abnormal seedlings were about 50%.

### 3.2. Effects of Nanopriming on Resolving Seed Dormancy

**Figure 5:**
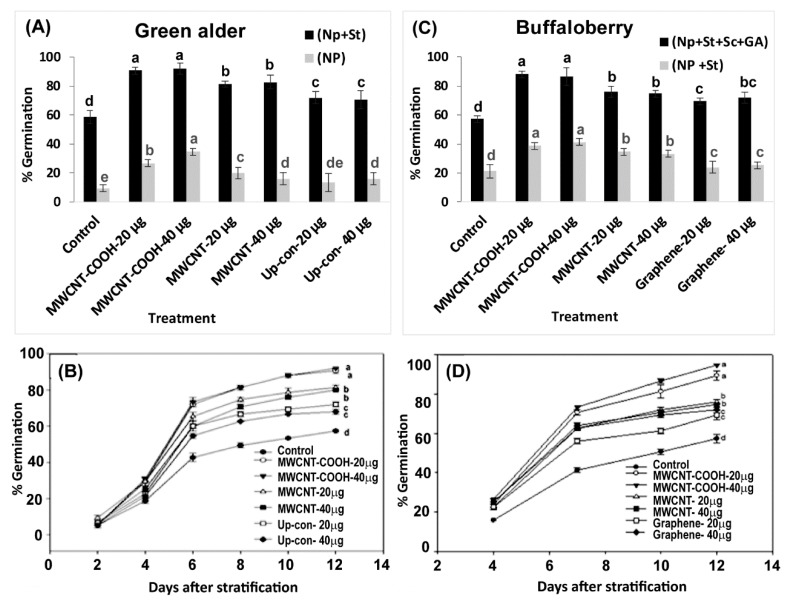
Effects of stratification and nanopriming treatments on seed germination percentage in green alder (**A**), buffaloberry (**B**) as well as temporal variations in germination percentage in green alder (**C**) and buffaloberry (**D**). Vertical bars or line graph represent germination percentage in green alder and buffaloberry ± standard errors. Different letters show significant differences among treatments at α = 0.05. Control = no CNPs added, MWCNT–COOH = multiwall carbon nanotubes functionalized with carboxylic acid, MWCNT = multiwall carbon nanotubes, UP-con NP = up conversion nanophosphors, N = 100 plants per treatment.

Seeds primed with MWCNT–COOH (40 µg/mL) had the highest germination compared to the control. This was approximately 35% higher than in the other nanoprimed treatments in green alder seeds (Figure 5A,B). However, MWCNT–COOH at 20 µg/mL) had slightly lower percent germination (30%) compared to MWCNT–COOH at (40 µg/mL), which was still higher compared to the other treatments (control, MWCNT and up conversion nano phosphorous). In the case of MWCNT and up conversion nano phosphorous at 20 µg/mL and 40 µg/mL, the germination rate was similar between both treatments (20%). In contrast, none of the buffalo berry seeds germinated following nanopriming without seed stratification.

Interestingly, when green alder seeds were primed with CNPs and cold stratified, higher germination was observed compared to when the seeds were only nanoprimed but not stratified. In all cases, the nanoprimed and cold stratified seeds had higher percent germination compared to the control. Among all the treatments, MWCNT–COOH (both concentrations) had the highest percent germination (90%). The germination percent was 80% in MWCNT (both concentrations), 70% in up conversion nano phosphorous (both concentrations), compared to 60% germination in the control (Figure 5A,B). The first green alder seed germinated after 2 days and attained a 90% rate of germination after 12 days in both concentrations of MWCNT–COOH treatments (Figure 5A,B).

On the other hand, buffaloberry seeds primed with CNPs and stratified showed marginal increase in total germination compared to the control, and the seeds that were only nanoprimed (not cold stratified) as shown in Figure 5C,D. Both concentrations of MWCNT–COOH had a higher percent germination (40%) compared to all other treatments. The seed germination ranged from 40% for MWCNT (20 µg/mL and 40 µg/mL), 35% for up-conversion nanoparticles and 30% for graphene (20 µg/mL and 40 µg/mL) (Figure 5C,D).

Due to unsatisfactory germination observed in buffaloberry seeds following nanopriming and cold stratification, we changed the germination treatment to include both mechanical and chemical scarification, as well as primed the seeds with plant hormone solutions (GA). We observed none of the seeds germinated in the chemical scarification treatments. In contrast, seeds mechanically scarified, cold stratified, and primed in both CNPs and GA solutions had significant increases in percent germination. Total germination was up to 90% in both MWCNT–COOH (20 µg/mL and 40 µg/mL) treated seeds. Similarly, a significantly higher germination was observed in seeds primed with MWCNT and graphene compared to the control, and ranged between 75% and 70%, respectively. The first buffaloberry seed germinated after 4 days and attained a 90% rate of germination 12 days in the MWCNT–COOH treatment (Figure 5D). Though germination started at the same time in both the control and nanoprimed seeds, generally, the number of seeds germinated were significantly higher at each time period point in the treated seeds compared to the control (Figure 5C,D).

### 3.3. Seedling Vigor Index

**Figure 6:**
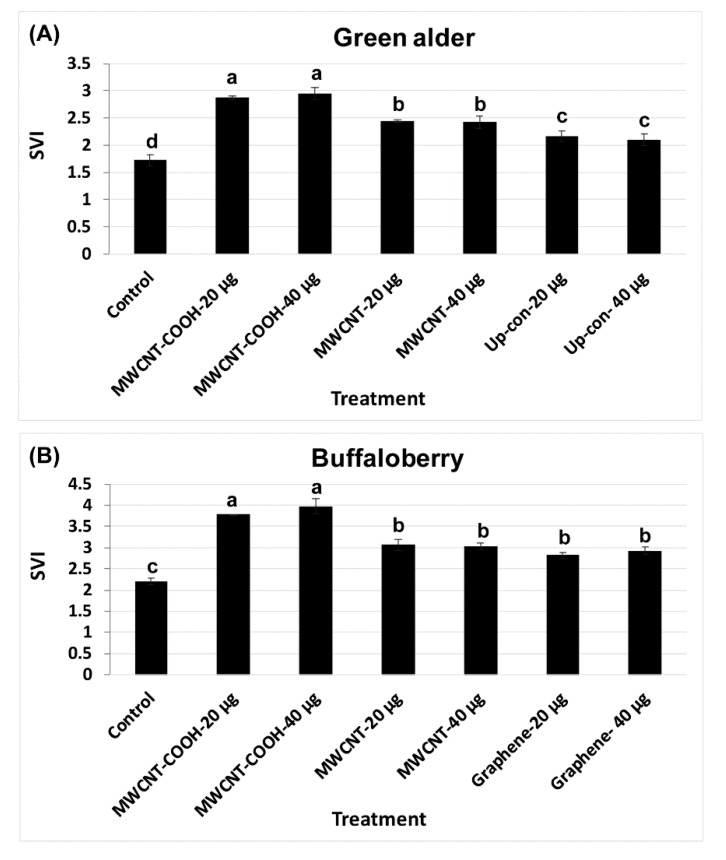
Seedling vigor index (SVI) in green alder (**A**) and buffalo berry (**B**) upland boreal forest species. Values in bar chart represent means ± standard errors and are significantly different at α = 0.05. Control = no CNPs added. MWCNT–COOH = multiwall carbon nanotubes functionalized with carboxylic acid, MWCNT = multiwall carbon nanotubes, up-con = up conversion nanophosphors. N = 100 plants per treatment.

CNPs treatments enhanced both seed germination and the seedling vigor index (SVI). The results show that SVI values in green alder primed with MWCNT–COOH (20 µg/mL and 40 µg/mL) were significantly (*p* = 0.004) higher (SVI = 2.8) than the control and other treatments (Figure 6A). SVI in seeds primed with MWCNT (20 µg/mL and 40 µg/mL), was 2.4, while in up conversion nano phosphorous (20 µg/mL and 40µg/mL) primed seeds, the SVI values were 2.2 and 2.1, respectively, compared to 1.6 in the control (Figure 6A). For buffalo berry, we measured seedling length, as well as the SVI for only mechanical scarification + stratification + CNPs + gibberellic acid treatments, as this combination of treatment showed the highest percent germination and germination rate (Figure 6B). MWCNT–COOH performed better than any other treatments at both concentrations evaluated (40 and 20 µg/mL). The SVI value was 4.00 and 3.75 respectively. Seeds stratified, scarified and primed in solutions of GA and MWCNT had similar SVI values (3.00) at both 40 and 20 µg/mL. In contrast, graphene treated seeds had higher SVI values at 40 µg/mL (2.90) than seeds primed at 20 µg/mL (2.80) (Figure 6A,B). The SVI in the control was lower than the nanoprimed treatments in both species and was 1.75 and 2.25 for green alder and buffalo berry, respectively (Figure 6A,B).

### 3.4. Roles of Lipid Metabolism in Overcoming Seed Dormancy in Upland Boreal Forest Species

**Figure 7:**
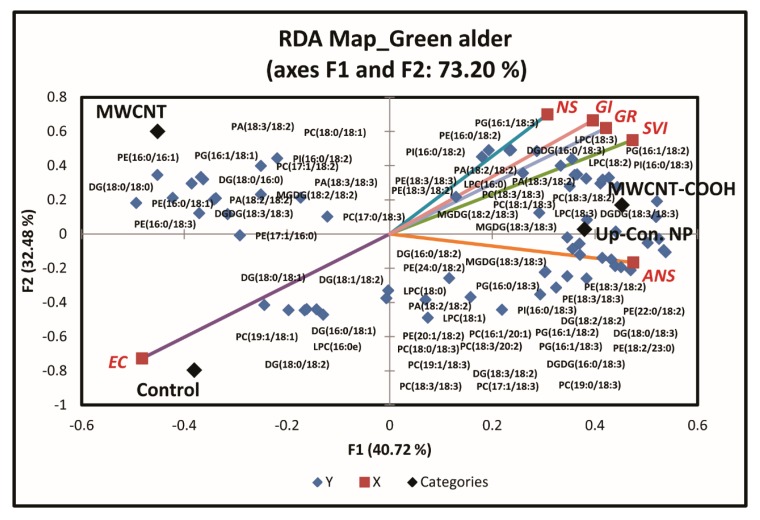
First redundancy analysis (RDA) of green alder showing the groupings of membrane lipids with seed physiological parameters following seed priming with CNP, where n = 100 plants for each treatment. MWCNT–COOH = multiwall carbon nanotubes functionalized with carboxylic acid, MWCNT = multiwall carbon nanotubes, up-con = up conversion nanophosphors. SVI = seedling vigor index, EC = electrical conductivity, GR = germination rate, NS = normal seedlings, ANS = abnormal seedlings.

Plant membrane lipids were evaluated to determine whether seed membrane lipid metabolism played a role in seed dormancy resolution following priming with nanoparticles. Approximately 12 membrane lipid classes and associated molecular species were quantitatively identified in both upland species. These include cardiolipin (CL), lysophosphatidylcholine (LPC), LPE (lysophosphatidylethnolamine), PA (phosphatidic acid), PC (phosphatidylcholine), PE (phosphatidylehanolamine), PG (phosphatidylglycerol), PI (phosphatidylinositol), PS (phosphatidylserine), DGDG (digalactosyldiacylglycerol), MGDG (monogalactosyl diglyceride), and SQDG (sulfoquinovosyl diacylglycerols). RDA analysis was conducted to discern the effects of the treatments on seedling membrane lipid composition. Seedlings with similar membrane lipids clustered in the same quadrant of the biplot (Figure 7 and Figure S1).

For buffaloberry, we analysed the lipidome for only the treatments of mechanical scarification + stratification + CNPs + gibberellic acid with 20 µg/mL concentration for all treatments, as there was no difference between both 20 µg/mL and 40 µg/mL concentrations in the seedling vigor index, germination rate, or normal and abnormal seedlings in any of the treatments (Figure S1). PC17:0/18:2, PS18:2/18:2, PC16:0/18:3, PS18:3/18:2, PE16:0/18:3, PC16:0/18:2, LPE18:0, LPC16:0, and PI16:0/18:2 clustered in Q1 with the MWCNT treatment. PE16:0/16:0, PS17:1/18:2, PE19:1/ 16:0, PE17:1/16:0, PE16:0/16:1, PE16:0/18:1, LPE16:0, PA18:2/18:2, PG16:0/16:0, PE16:1/18:1, PC15:0/18:2, and PA18:1/18:2 were clustered in Q2 with the Graphene treatments. MGDG18:3/18:3, DGDG18:0/18:3, LPC18:2, DGDG18:3/18:3, PG16:1/18:3, PI16:0/18:2, PC18:1/18:3, LPE18:2, PC18:3/18:2, PA18:3/18:2, PE18:2/18:2, PE18:3/18:2, LPC18:3, PA18:3/18:3, PS18:2/18:2, SQDG16:0/18:1, PI16:0/18:3, and PG16:1/18:2 were clustered in Q3 with the MWCNT-COOH treatment, along with germination rate, normal seedlings, and SVI. LPC18:0, LPC18:1, PG16:0/16:1, PE15:0/18:2, PA18:0/18:2, PC16:0/18:1, PC18:0/18:1, PC18:0/18:2, PI16:0/18:1, PC18:1/18:2, and PI16:0/18:3 were clustered in Q4 with the control and electrical conductivity. Approximately, 73.7% of the total variability in the data accounted for this segregation (Figure S1).

For green alder (Figure 7), we only evaluated the membrane lipids in CNPs + stratification treatment at 20 µg/mL concentrations for the same reason as with buffaloberry. PG16:1/18:1, DG18:0/18:0, PI16:0/18:2, PC17:1/18:2, PA18:3/18:2, PE16:0/16:1, DG18:0/16:0, PA18:3/18:3, PE16:0/18:1, PA18:2/18:2, MGDG18:2/18:2, PC18:0/18:1, DGDG18:3/18:3, PE16:0/18:3, and PC17:0/18:3 lipid classes were clustered in Q1 with the MWCNT treatment. MGDG18:3/18:3, PC18:3/18:3, LPC18:3, PE18:3/18:2, DGDG16:0/18:3, PG16:1/18:2, LPC18:2, PE18:3/18:3, PI16:0/18:2, PI16:0/18:3, PA18:3/18:2, PA18:2/18:2, DGDG18:3/18:3, LPC16:0, PC18:3/18:2, PG16:1/18:3, PC18:1/18:3, PE16:0/18:2, LPC18:3, MGDG18:2/18:3, and PC19:0/18:3 lipid classes were clustered in Q2 with the MWCNT–COOH treatment, normal seedlings, germination rate, and seedling vigor index. In Q3, LPC16:0e, DG16:0/18:1, DG18:0/18:1, DG18:0/18:2, DG18:1/18:2, and PC19:1/18:1 lipid classes were clustered with the control and electrical conductivity. DG16:0/18:2, PE24:0/18:2, PA18:2/18:2, DGDG16:0/18:3, PE18:3/18:3, PC8:3/18:3, MGDG18:3/18:3, PC17:1/18:3, LPC18:0, PC16:1/20:1, PC18:0/18:3, PG16:0/18:3, PE20:1/18:2, LPC18:1, PC19:1/18:3, DG18:0/18:3, DG18:2/18:2, PG16:1/18:2, PG16:1/18:3, DG18:3/18:2, PE22:0/18:2, PE18:3/18:2, PE18:2/23:0, and PC18:3/20:2 lipid classes were clustered in Q4 with the MWCNT treatment and abnormal seedlings. Approximately, 73.2% of the total variability in the data accounted for this segregation (Figure 7).

In the first RDA, quadrant three (Q3) clustered most of the physiological parameters of the seedlings (germination rate, normal and abnormal seedlings, seedlings vigor index) along with the MWCNT–COOH treatment and the associated lipid molecular species for buffaloberry (Figure S1). Considering the majority of the physiological parameters clustered with MWCNT–COOH, a second RDA was conducted using only the lipid classes/molecular species from Q3 of the RDA plot of MGDG18:3/18:3, DGDG18:0/18:3, LPC18:2, DGDG18:3/18:3, PG16:1/18:3, PI16:0/18:2, PC18:1/18:3, LPE18:2, PC18:3/18:2, PA18:3/18:2, PE18:2/18:2, PE18:3/18:2, LPC18:3, PA18:3/18:3, PS18:2/18:2, SQDG16:0/18:1, PI16:0/18:3, and PG16:1/18:2 to further refine the association between the treatments, altered lipid metabolism, seed germination, and SVI (Figure S2).

**Figure 8:**
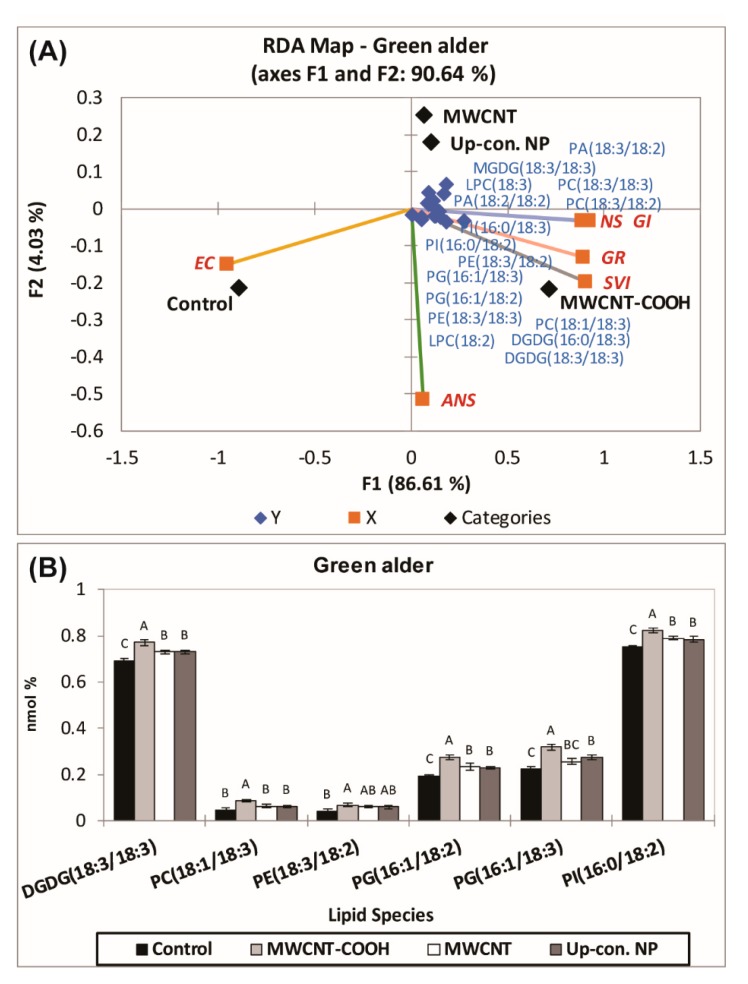
(**A**) Second RDA for those lipid classes which clustered with MWCNT–COOH treatments during first RDA in green alder (**A**). ANOVA of the lipid species found in green alder which were clustered in Q4 with the MWCNT–-COOH treatment (**B**). Values in bar chart represent means ± standard errors and all are significantly different at α = 0.05, n = 100 plants per treatment. MWCNT–COOH = multiwall carbon nanotubes functionalized with carboxylic acid, up con np = up conversion nanophosphors. SVI = seedling vigor index, EC = electrical conductivity, GR = germination rate, NS = normal seedlings, ANS = abnormal seedlings.

In the case of green alder (Figure 7), a second RDA was also conducted with the lipid classes of Q2 (Figure 8A), including MGDG18:3/18:3, PC18:3/18:3, LPC18:3, PE18:3/18:2, DGDG16:0/18:3, PG16:1/18:2, LPC18:2, PE18:3/18:3, PI16:0/18:2, PI16:0/18:3, PA18:3/18:2, PA18:2/18:2, DGDG18:3/18:3, LPC16:0, PC18:3/18:2, PG16:1/18:3, PC18:1/18:3, PE16:0/18:2, LPC18:3, MGDG18:2/18:3, and PC19:0/18:3, clustered with the MWCNT–COOH treatment, as most of the physiological parameters clustered in this quadrant (Figure 8A).

Following the second RDA in buffaloberry (Figure S2), the following lipids [DGDG18:0/18:3, SQDG16:0/18:1, PE18:3/18:2, PS18:2/18:2, LPC18:2, PC18:3/18:2, PC18:1/18:3, PA18:3/18:2, and PI16:0/18:3] were clustered in Q2 with the MWCNT–COOH treatment rather than with the MWCNT, control, or graphene. In case of green alder, [PI16:0/18:2, PG16:1/18:3, PE18:3/18:2, DGDG18:3/18:3, DGDG16:0/18:3, LPC18:2, PC18:1/18:3, PE18:3/18:3, and PG16:1/18:2] were clustered in Q4 with the MWCNT–COOH treatment. Those clusters were then used to determine the relationship between SVI, germination rate, EC, normal and abnormal seedlings. It was observed from the second RDA map that SVI, germination rate, and normal and abnormal seedlings clustered with the MWCNT–COOH treatment, and EC clustered with the control in both upland species. Approximately 72.56% (Figure S2) and 90.64% (Figure 8A) variability in the data accounted for the segregation of buffaloberry and green alder, respectively, in the second RDA analysis.

**Figure 9:**
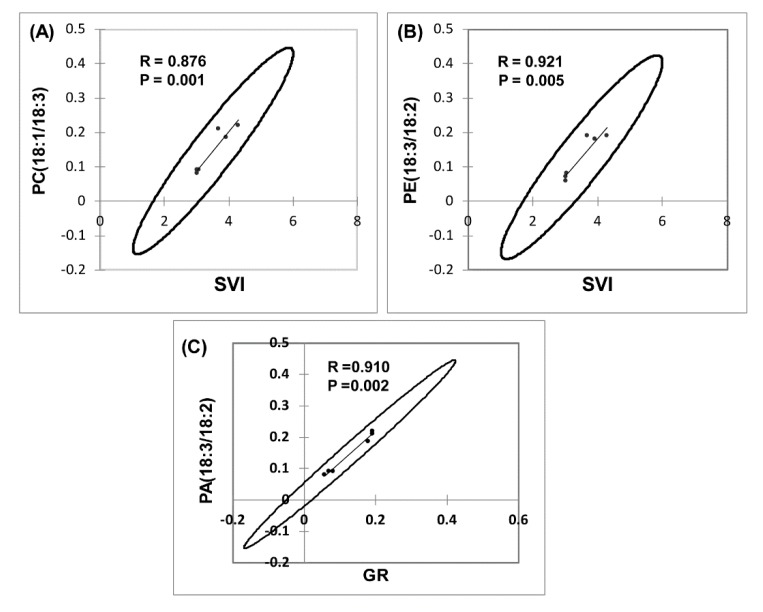
Correlation of lipid molecular species with seedling vigor index (SVI) and germination rate (GR) in dormant upland boreal forest species. Correlation between PC(18:1/18:3) and SVI (**A**), PE(18:3/18:2) and SVI (**B**) and PC(18:3/18:2) and GR (**C**). Means of the treatments used for MWCNT–COOH are presented, N = 100 plants per treatment. PC = phosphatidylcholine, PA = phosphatidic acid, PE = phosphotidylethanolamine.

ANOVA was employed to examine the effects of the lipid molecular species that clustered GR, SVI, and NS, with MWCNT–COOH (the best performing treatment). For buffaloberry, PI16:0/18:2, PE18:3/18:2, PC18:1/18:3, PA18:3/18:2, and for green alder, PI16:0/18:2, PG16:1/18:3, PG16:1/18:2, PE18:3/18:2, PC18:1/18:3, DGDG18:3/18:3 levels were elevated in the MWCNT–COOH treatment compared to the controls (Figure 9 and Figure S2). Among all the lipid classes observed to be elevated with MWCNT–COOH after ANOVA, only PC18:3/18:3 and PE18:3/18:2 were observed to be common in both upland species. The lipid molecular species segregated with MWCNT–COOH was then correlated with seedling vigor index and total seed germination using Pearson correlation coefficient to assess the strength of the association between these lipid molecular species, seedling vigor and seed germination. We observed PC18:1/18:3 and PE18:3/18:2 were positively correlated with the SVI (*R* = 0.876, *p* = 0.001 and *R* = 0.921 and *p* = 0.005 respectively) (Figure 9A,B), while PA18:3/18:2 was strongly correlated with GR (*R* = 0.910 and *p* = 0.002) (Figure 9C). The other lipid molecular species that clustered in the same quadrants as SVI and GR following RDA analysis were not significantly correlated with any of these seed physiological parameters (SVI and GR) following Pearson correlation analysis (Supplementary Tables S1 and S2).

## 4. Discussion

### 4.1. Effect of Carbon Nanoparticles on Seedling Quality

The tetrazolium test is employed to measure seed germination and its viability [61,62]. In instances where seeds are impermeable, that is, when the coats make it hard to absorb nutrients, other test mechanisms (germination rate, normal or abnormal seedlings, and germination index) are adopted to assess viability [63]. We observed approximately 90% of the seeds were viable for both buffalo berry and green alder following nanopriming (Figure 1). The recording of 90% viability for both tested species means that the seed lots have the potential to enhance plant production (Figure 1C) and that the seeds used for further study evaluations were viable.

Higher EC readings observed in the control primed with DI compared to the nanoprimed seeds (Figure 2), indicated nanopriming improved the integrity of the seed cell membrane, thereby reducing electrolyte leakage from the cell membrane. This contributed to seeds with improved seed vigor compared to hydro primed seeds (control treatment) [16,23,64]. Previous research findings showed that seed membrane integrity has a direct effect on seed viability and seedling quality [64,65].

Approximately 85% of the primed seeds were normal, indicating that nanopriming improved boreal forest seedling quality. Seed quality is instrumental in the production of seedlings for agriculture and forestry sectors. Trueness to variety, germination percentage, purity, seedling vigor, and appearance are important characteristics of seed quality [49]. Achieving and maintaining high seed and seedling quality is very important for successful plant propagation. Our findings revealed MWCNT–COOH was the best performing treatment regarding the production of normal seedlings for both upland forest species evaluated in this study, and that a concentration of 20 µg/mL was highly effective (Figure 3 and Figure 4). According to our findings, nanopriming improved seed viability and the ability of seedlings to germinate and potentially be established across a range of environmental sites (improved seedling vigor). This finding is also consistent with previous reports that nanopriming with CNPs can be used to increase seedling production and seed quality parameters [34,66]. For example, phosphorylation efficiency has been demonstrated to be restricted by poor membrane integrity, resulting in low germination rates [67]. It is possible that one-way nanopriming contributes to superior seed germination, and SVI measures may be associated with improved phosphorylation efficiency in the seeds through improvement in the seed cell membrane integrity during the resolution of seed dormancy. This is a subject for future studies to better understand, how nanopriming with CNPs may be working to mechanistically alleviate seed dormancy in upland boreal forest species through improved phosphorylation efficiencies.

### 4.2. Effects of Carbon Nanoparticles in Resolving Seed Dormancy in Upland Boreal Forest Species

Buffaloberry has a hard seed coat (*physical dormancy*), which impedes germination [9,10], as the seed coats are impermeable to water and other nutrients, ultimately preventing the germination of viable seeds [39,68,69]. On the other hand, green alder is inhibited by embryo dormancy [12,70], which is a second category dormancy (morphophysiological dormancy). This dormancy results in low seed germination and stand establishment [70]. Cold-moist stratification has been demonstrated to be very effective in breaking seed dormancy and improving seed germination rate and seedling development [37,39,68,71]. The seeds primed with CNPs and stratified showed higher germination compared to the control seeds or when the seeds were primed without CNPs. The best increase in germination rate in both upland species was observed when the seeds were primed with MWCNT–COOH, suggesting that the application of functionalized CNPs might have more positive effects on germination and resolution of seed dormancy (Figure 6 and Figure S1).

The unfunctionalized MWCNTs increase the seed germination rate and plant growth by increasing the moisture content of seeds and enhance the water absorption machinery of root tissues [72]. It appears the modified CNTs (MWCNT–COOH) may have enhanced the moisture contents in seeds by creating a thicker water layer around them. Furthermore, MWCNTs/or OMWCNTs can also be transported through the plant’s vascular cylinder. Many studies showed an effect of non-functionalized MWCNT on plants (germination, seedlings growth, seed vigor, etc.), but limited research has been conducted on MWCNT functionalized with –COOH. In this study, we used MWCNT functionalized with –COOH to successfully resolve both embryo and seed coat dormancy, as well as improve seed germination in two upland boreal forest species.

### 4.3. Effect of Carbon Nanoparticles in Enhancing Seedling Vigor

Concomitant with the low seed germination in these species, due to dormancy issues, the germinated seedlings can also have low vigor or growth rates [12]. The seedling vigor index (SVI) can be measured through seedling vigor test [73,74,75] and it is very important to have seedlings with enhanced vigor, or SVI. Seedlings with enhanced SVI have an increased chance of survival and establishment across different environments with varying stressors, particularly boreal forest sites required to be revegetated following resource mining [70,76]. These sites have many challenges (water pollution, high bitumen content, nutrient deficiency, etc.). The results from our studies showed that the seedlings obtained following nanopriming had increased root and shoot length compared to the control seedlings. The increase seedling growth rate may be due to enhanced water and nutrient uptake capacity by the CNPs treated seeds [33,42,77]. In fact, tomato seeds incubated in agar medium supplemented with CNTs absorbed the CNTs inside the seeds resulting in seeds with a high percentage of moisture [78]. This maybe the mechanism through which CNPs contribute to improved shoot and root growth following nanopriming [79]. The observation that CNPs enhanced the seedling vigor index indicates that these seeds have enhanced capacity to be able to germinate, get establish, and survive under a range of challenging or stressful environmental conditions. This means that CNPs have the potential to enhance the vigor of native boreal forest seeds during propagation to facilitate plant growth under varied environments and may have applications in boreal forest restoration or reclamation programs [7,8,12].

In our study, MWCNT–COOH increased the seedling vigor of both buffalo berry and green alder, indicating that a greater effect on root and shoot length development, compared to the control and other nanoprimed treatments. However, in buffalo berry, the SVI value was higher than that observed in green alder (Figure 6). Others have reported that the effectiveness of the application of CNTs may depend on the plant species. Cañas et al., [29] studied the effect of functionalized SWCNTs (with poly-3-aminobenzenesulfonic acid for higher dispersal ability) and non-functionalized SWCNTs on the root growth of six crop species: cabbage (*Brassica oleracea* L.), carrot (*Daucus carota* L.), cucumber (*Cucumis sativus* L.), lettuce (*Lactuca sativa* L.), onion (*Allium cepa* L.), and tomato (*Solanum lycopersicum* L.). Their results indicate that the effect of applying CNTs differed, depending on the species, and this effect was also observed in our study. Enhanced water and ionic nutrient uptake are stimulated using CNTs, explaining perhaps why growth is then stimulated, as discovered for maize (*Zea mays* L.) [80].

### 4.4. Role of Lipid Metabolism in Resolving Seed Dormancy in Upland Boreal Forest Species

**Figure 10:**
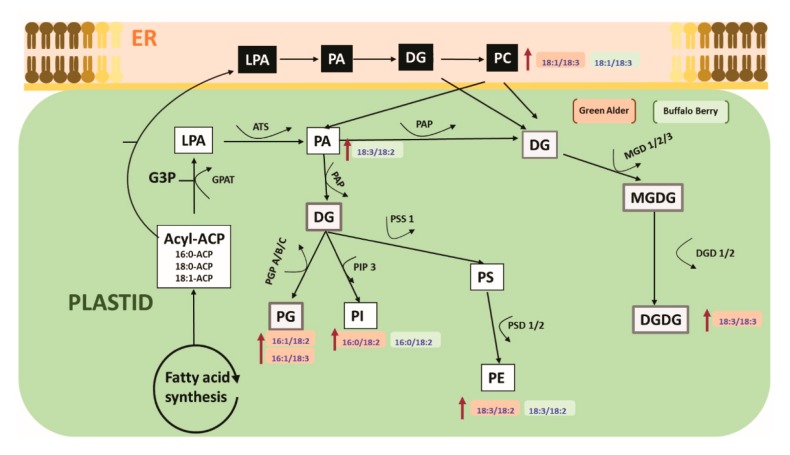
Proposed pathway showing the effects of nanopriming with MWCNT–COOH on the membrane lipid metabolism of upland boreal forest species in response to resolving seed dormancy ER = endoplasmic reticulum, LPA = lysophosphatidic, PA = phosphatidic acid, DG = diacylglycerol, PC = phosphatidylcholine, PG = phosphatidyl glycerol, PI = phosphatidylinositol, PS = phosphatidylserine, PE = phosphatidylehanolamine, MGDG = monogalactosyldiacylglycerol, DGDG = digalactosyldiacylglycerol, PGP = glycerol-3-phosphate phosphatase, EPT = ethanolamine phosphotransferase, DGD = dialkylglycine decarboxylase, PAP = phosphatidic acid phosphatase, MGDG = monoalkylglycine decarboxylase, PSD = phosphatidylserine decarboxylase, PIP 3 = 1-phosphatidylinositol-4-phosphate 5-kinase, ATS = acetyl hydrolase.

A limited number of studies have shown that alterations in cell membrane lipids may be involved in the alleviation of seed dormancy in agriculture crop species [45,46,81]. For example, during germination seed membrane lipids were observed to be remodelled by the gradual increase of plastidic lipids, where the level of PA first decreased followed by an elevation (increase) before finally decreasing in soybeans [79,80,81,82]. Changes in seed fatty acids have also been reported to be directly associated with alleviating seed embryo dormancy, and a remarkable change in linoleic acid levels in *Amaranthus albus* seeds before and after breaking dormancy was observed to be associated with the reported success [51]. Although, these reports indicate the seed membrane lipidome may play a role during the alleviation of seed dormancy in some agriculture crop species, there is no report to our knowledge documenting the role of lipid metabolism in alleviating seed dormancy in native boreal forest species. In this study, we show for the first time that upland boreal forest species primed and stratified with MWCNT functionalized with carboxylic acid (MWCNT–COOH) was very effective in improving germination and seedling vigor in dormant buffalo berry and green alder seeds (Figure 4 and Figure 5, Figures S1 and S2). Elevated levels of PI16:0/18:2, PE18:3/18:2, PC18:1/18:3 and PA18:3/18:2 were observed to be significantly correlated with resolution of seed dormancies in these species (Figure 8 and Figure S2). These molecular species are related biosynthetically, as shown in the proposed lipid metabolism pathway (Figure 10). PI, PE and PA synthesis occur in the plastid, but PC synthesis occurs in the endoplasmic reticulum. PI16:0/18:2 is synthesized from DG by phosphatidylinositol-4-phosphate-5-kinase. PE18:3/18:2 is synthesized from PS by phosphatidylserine decarboxylases (PSD1/2). PA18:3/18:2 is synthesized from LPA by acetyl hydrolase (ATS). PC18:3/18:3 is synthesized from DG by CDP choline transferase. It thus appears that priming dormant buffalo berry and green alder seeds with MWCNT–COOH appears to elevate the activities of these enzymes thereby contributing to the elevated molecular species that were significantly correlated with the improved germination and seedling vigor observed in this study. Furthermore, C18:3 and C18:2 enriched PI, PE, PC and PA molecular species appear to be enhanced in buffaloberry and green alder seeds in response to the treatments with MWCNT–COOH. In the proposed metabolic pathway, the increase in C18:3 enriched molecular species suggest that *∆3* desaturase seems to play a vital role in modulating the membrane lipids during the resolution of seed dormancy and the increase seedling vigor observed in upland boreal forest species (green alder and buffaloberry). 

The purpose of using –COOH functionalized CNTs is to best match up the biochemistry of the plant with the chemistry of the CNTs. –COOH represents the functional group of a carboxylic acid which tend to be esterified to a hydrocarbon in the membrane structure of all living cells. Fatty acids are the monomers for all lipids that are essential biomolecules in cellular metabolism. As such, we hypothesized that the carboxylic acid functionalized CNTs would be more effective in modulating the seed membrane lipidome to effectively resolve seed dormancy. Seed lipid metabolism is recognized as an important response determining successful germination in all plant species. In this study, it appears –COOH functionalized CNTs were more effective in modulating the biosynthesis of C18:3 enriched PC, PE and PA molecular species which appears to be important in the resolution of seed dormancy in upland boreal forest species.

Recently, nanoparticles have been shown to modulate lipid metabolism in plants [83,84]. Martinez-Ballesta et al., [85] stated that nanopriming with CNPs increased the aquaporins, ion, and water transportation in cell membranes. Also, these authors found that CNPs helped in maintaining electrostatic or homeostasis balance in cell membranes and that the membrane forms new lipid domains, or raft, in broccoli, as a consequence of the CNPs induced lipid metabolism. Our findings demonstrated that that carboxylic acid functionalized MWCNT altered the seed membrane lipid metabolism in dormant seeds having both seed coat and embryo dormancies. The resultant improved germination and seedling vigor were observed to be highly correlated with the altered lipid species shown in the proposed lipid biosynthetic pathway (Figure 10). Specifically, the biosynthetic routes involving PA, DG, PC and PE seems to be modulated by MWCNT–COOH during the alleviation of seed coat and embryo dormancies, resulting in improved germination and SVI in upland boreal forest species using bog birch and green alder as test plants.

## 5. Conclusions

This study aimed to understand how nanotechnologies could be applied to improve seed germination by overcoming seed dormancy in native upland boreal forest species. The findings in this study demonstrate that priming seeds with a 20 µg/mL concentration of MWCNT–COOH and GA along with stratification at two to four degrees was very effective in resolving seed dormancy in two upland boreal forest species: green alder and buffalo berry. Resolution of seed dormancy occurred concomitant with increases in germination and SVI. The improved germination and SVI were observed to be clustered with several membrane lipid molecular species enriched with C18:3 fatty acids when nanoprimed with MWCNT–COOH. These lipids (PE18:3/18:2, PC18:1/18:3, and PA18:3/18:2) were observed to be significantly elevated compared to the control and to be highly correlated with seed germination and the seedling vigor index. These results indicate that MWCNT functionalized with carboxylic acids may be useful in resolving seed dormancy in upland boreal forest species by modulating cell membrane lipid metabolism. The proposed metabolic pathway of the membrane lipids showed that C18:3 enriched fatty acids play a key role in alleviating seed coat and embryo dormancy in the tested upland boreal forest species (buffaloberry and green alder). Nanopriming seeds with low concentrations of MWCNT–COOH could be useful in improving the propagation by seeds of non-resource boreal forest species with seed dormancy issues, and may have applications in propagating these species for use in boreal forest reclamation or restoration following resource extraction and/or anthropogenic disturbances. Furthermore, the seedlings obtained have improved SVI, indicating an enhanced potential to be established across a range of sites with varying environmental conditions. Currently, there are significant challenges in the forest reclamation industry to successfully propagate many native boreal forest species with dormant seeds required to meet boreal forest reclamation standards. The findings from this study may be useful to this sector and improve knowledge and understanding in the scientific community of how nanopriming can successfully alleviate seed dormancy in native boreal forest species through modulation of the cell membrane lipidome.

## Supplementary Materials

The following are available online at https://www.mdpi.com/2079-4991/10/1/176/s1, Figure S1: First Redundancy analysis (RDA) of buffaloberry showing the groupings of membrane lipids with seed physiological parameters following seed priming with CNP, where n = 100 plants for each treatment. MWCNT–COOH = multiwall carbon nanotubes functionalized with carboxylic acid; MWCNT= multiwall carbon nanotubes, up-con = up conversion nanophosphorus. SVI = seedling vigor index, EC = electrical conductivity, GR = germination rate, NS = normal seedlings, ANS = abnormal seedlings, Figure S2: Second RDA for those lipid classes which clustered with MWCNT–COOH treatments during first RDA in buffaloberry. Values in bar chart represent means ± standard errors and all are significantly different at α = 0.05, n = 100 plants per treatment. MWCNT–COOH = multiwall carbon nanotubes functionalized with carboxylic acid, SVI = seedling vigor index, EC = electrical conductivity, GR = germination rate, NS = normal seedlings, ANS = abnormal seedlings, Table S1: Lipid molecular species clustered in the same quadrants as SVI and GR following RDA analysis but are not significantly correlated following Pearson correlation analysis in buffaloberry. N = 100, plants per treatment, Table S2: Lipid molecular species clustered in the same quadrants as SVI and GR following RDA analysis but are not significantly correlated following Pearson correlation analysis in green alder. N = 100 plants per treatment.
