# Supplementary material for: Carbon Nanoparticles Functionalized with Carboxylic Acid Improved the Germination and Seedling Vigor in Upland Boreal Forest Species
Source: Nanomaterials (Basel). 2020 Jan 20;10(1):176. doi: 10.3390/nano10010176 (PMC7023356; doi:10.3390/nano10010176)
Supplement: Supplementary file 1 [file nanomaterials-10-00176-s001.pdf]

Carbon Nanoparticles Functionalized with Carboxylic Acid Improved the Germination and Seedling Vigor in Upland Boreal Forest Species

Md. Hossen Ali [1], Jean-Marie Sobze [2], Thu Huong Pham [1], Muhammad Nadeem [1,*], Chen Liu [1], Lakshman Galagedara [1], Mumtaz Cheema [1] and Raymond Thomas [1,*]

[1] School of Science and the Environment/Boreal Ecosystem Research Facility, Grenfell Campus, Memorial University of Newfoundland, Corner Brook, A2H 5G5, Canada; mhali@grenfell.mun.ca (M.H.A.); tpham@grenfell.mun.ca (T.H.P.); cliu@grenfell.mun.ca (C.L.); lgalagedara@grenfell.mun.ca (L.G.); mcheema@grenfell.mun.ca (M.C.)

[2] Northern Alberta Institute of Technology, 8102–99 Avenue, Peace River, Alberta T8S 1R2, Canada; jeanmars@nait.ca

* Correspondence: mnadeem@grenfell.mun.ca (M.N.); rthomas@grenfell.mun.ca (R.T.)

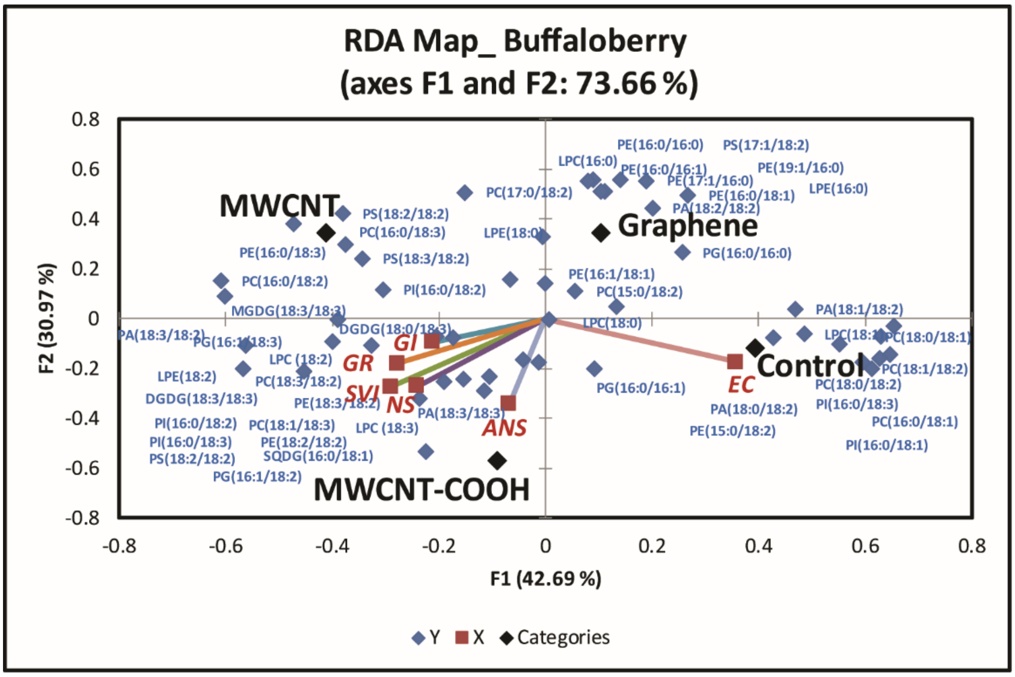

Figure S1. First Redundancy analysis (RDA) of buffaloberry showing the groupings of membrane lipids with seed physiological parameters following seed priming with CNP, where n = 100 plants for each treatment. MWCNT–COOH = multiwall carbon nanotubes functionalized with carboxylic acid; MWCNT= multiwall carbon nanotubes, up-conversion = up conversion nanophosphorus. SVI = seedling vigor index, EC = electrical conductivity, GR = germination rate, NS = normal seedlings, ANS = abnormal seedlings.

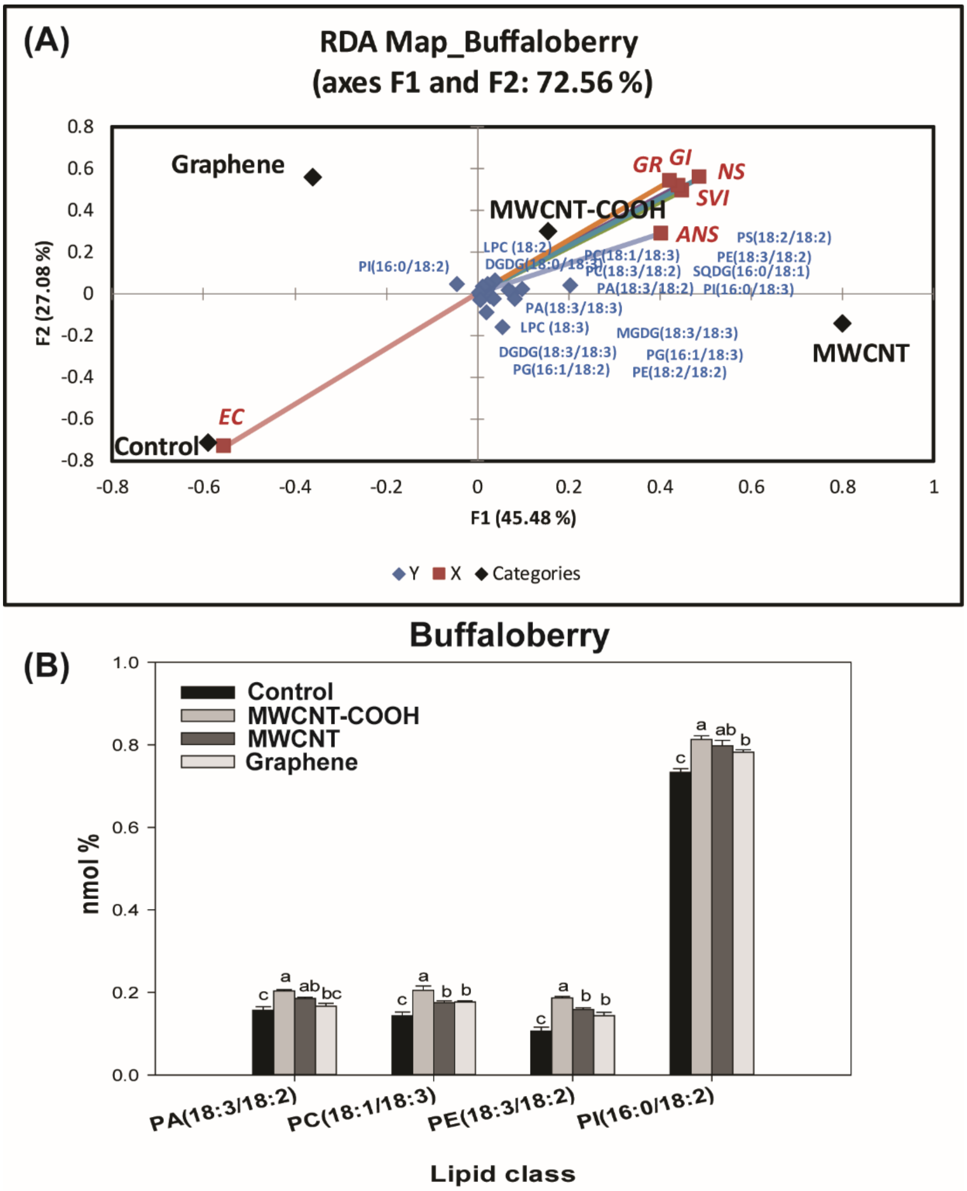

Figure S2. Second RDA for those lipid classes which clustered with MWCNT–COOH treatments during first RDA in buffaloberry. Values in bar chart represent means ± standard errors and all are significantly different at $\alpha = 0.05$, n = 100 plants per treatment. MWCNT–COOH = multiwall carbon nanotubes functionalized with carboxylic acid, SVI = seedling vigor index, EC = electrical conductivity, GR = germination rate, NS = normal seedlings, ANS = abnormal seedlings.

Table S1. Lipid molecular species clustered in the same quadrants as seedling vigor index (SVI) and germination rate (GR) following RDA analysis but are not significantly correlated following Pearson correlation analysis in buffaloberry. N = 100 plants per treatment.

**Buffaloberry**	
**Name of lipid classes**	**Level of significance**
LPC (18:2)	NS
PC (18:3/18:2)	NS
PI (16:0/18:3)	NS
PS (18:2/18:2)	NS
DGDG (18:0/18:3)	NS
SQDG (16:0/18:1)	NS

Table S2. Lipid molecular species clustered in the same quadrants as seeding vigor index (SVI) and germination rate (GR) following RDA analysis but are not significantly correlated following Pearson correlation analysis in green alder. N = 100 plants per treatment.

**Green alder**	
**Name of lipid classes**	**Level of significance**
DGDG (16:0/18:3)	NS
LPC (18:2)	NS
PE (18:3/18:3)	NS
DGDG (18:3/18:3)	NS
PI (16:0/18:2)	NS
PG (16:1/18:3)	NS
PG (16:1/18:2)	NS
